# Tolerogenic Dendritic Cells as a Promising Antigen-Specific Therapy in the Treatment of Multiple Sclerosis and Neuromyelitis Optica From Preclinical to Clinical Trials

**DOI:** 10.3389/fimmu.2018.01169

**Published:** 2018-05-31

**Authors:** Georgina Flórez-Grau, Irati Zubizarreta, Raquel Cabezón, Pablo Villoslada, Daniel Benitez-Ribas

**Affiliations:** ^1^Department of Immunology, Hospital Clinic i Provincial, Barcelona, Spain; ^2^Neuroimmunology Group, Institut d’Investigacions Biomèdiques August Pi i Sunyer (IDIBAPS), Barcelona, Spain

**Keywords:** tolerogenic dendritic cells, dendritic cells, immunotherapy, immunosuppression, multiple sclerosis, Neuromyleitis optica

## Abstract

The identification of activated T-lymphocytes restricted to myelin-derived immunogenic peptides in multiple sclerosis (MS) and aquaporin-4 water channel in neuromyelitis optica (NMO) in the blood of patients opened the possibility for developing highly selective and disease-specific therapeutic approaches. Antigen presenting cells and in particular dendritic cells (DCs) represent a strategy to inhibit pro-inflammatory T helper cells. DCs are located in peripheral and lymphoid tissues and are essential for homeostasis of T cell-dependent immune responses. The expression of a particular set of receptors involved in pathogen recognition confers to DCs the property to initiate immune responses. However, in the absence of danger signals different DC subsets have been revealed to induce active tolerance by inducing regulatory T cells, inhibiting pro-inflammatory T helper cells responses or both. Interestingly, several protocols to generate clinical-grade tolerogenic DC (Tol-DC) *in vitro* have been described, offering the possibility to restore the homeostasis to central nervous system-related antigens. In this review, we discuss about different DC subsets and their role in tolerance induction, the different protocols to generate Tol-DCs and preclinical studies in animal models as well as describe recent characterization of Tol-DCs for clinical application in autoimmune diseases and in particular in MS and NMO patients. In addition, we discuss the clinical trials ongoing based on Tol-DCs to treat different autoimmune diseases.

## Introduction

Multiple Sclerosis (MS) is a chronic autoimmune inflammatory disease affecting the central nervous system (CNS) (1). Nowadays, there are 2.3 million affected people worldwide, being the most frequent age of diagnosis between 20 and 40 years old (2). Additionally, the studies determine that MS is more frequent in women and in northern locations. There are different subtypes of MS which are based on their clinical phenotype (3). These subtypes are: The primary-progressive MS (PPMS) which is a disabling subtype from the beginning, the relapsing-remitting type (RRMS) that is characterized by clinical relapses without progression of disability and finally, the secondary-progressive subtype that appears about 20 years after RRMS.

The MS diagnosis is summarized in the revised 2010 Mc Donald criteria which is included in Table 1 (4). Although the cause of the immune deregulation is unknown, there are evidences that implicate Th1 and Th17 lymphocytes in the pathophysiology of MS (5–10). Furthermore, it was supported by studies performed in experimental models of MS either knocking out or blocking using monoclonal antibodies for IL-17 or IL-23 resulted in a suppression of the activity of this disease (11, 12). Other authors have described that memory T-cells are activated in the periphery by different processes that can be promoted by environmental or genetic factors. These activated cells cross the blood–brain barrier, penetrate to CNS where they are locally reactivated (9, 13).

**Table 1:** 2010 Mc Donald criteria for multiple sclerosis (MS) diagnosis (4).

Clinical presentation	Additional data needed for MS diagnosis
2 or more attacks; objective clinical evidence of 2 or more lesions or objective clinical evidence of 1 lesion with reasonable historical evidence of a prior attack	None

2 or more attacks; objective clinical evidence of 1 lesion	Dissemination in space, demonstrated by: 1 or more T2 lesions in at least 2 of 4 MS-typical regions of the central nervous system (CNS) (periventricular, juxtacortical, infratentorial, or spinal cord); or await a further clinical attack implicating a different CNS site

1 attack; objective clinical evidence of 2 or more lesions	Dissemination in time, demonstrated by: simultaneous presence of asymptomatic gadolinium-enhancing and non-enhancing lesions at any time; or A new T2 and/or gadolinium-enhancing lesion(s) on follow-up MRI, irrespective of its timing with reference to a baseline scan; or await a second clinical attack

1 attack; objective clinical evidence of 1 lesion (clinically isolated syndrome)	Dissemination in space and time, demonstrated by: for DIS: 1 or more T2 lesion in at least 2 of 4 MS-typical regions of the CNS (periventricular, juxtacortical, infratentorial, or spinal cord); or await a second clinical attack implicating a different CNS site; and for DIT: simultaneous presence of asymptomatic gadolinium-enhancing and non-enhancing lesions at any time; or a new T2 and/or gadolinium-enhancing lesion(s) on follow-up MRI, irrespective of its timing with reference to a baseline scan; or Await a second clinical attack

Insidious neurological progression suggestive of MS (PPMS)	1 year of disease progression (retrospectively or prospectively determined) plus 2 of 3 of the following criteria: evidence for DIS in the brain based on 1 or more T2 lesions in the MS-characteristic (periventricular, juxtacortical, or infratentorial) regions evidence for DIS in the spinal cord based on 2 or more T2 lesions in the cord positive CSF (isoelectric focusing evidence of oligoclonal bands and/or elevated IgG index)

First-line therapies for MS include injectable treatments such as IFN-β, and glatiramer as well as oral therapies such as teriflunomide and dimethyl-fumarate. Second-line therapies include fingolimod, and the intravenous natalizumab, which present higher levels of efficacy in reducing the relapse rate; however, it has potential severe side effects. Moreover, Alentuzumab, Cladribine, and Ocrelizumab were recently added as approved therapies, and they are in progress of being defined in the pyramid of the MS therapy. All these mentioned treatments are systemic immunomodulatory or immunosuppressive treatments with risks of adverse events.

Neuromyelitis optica (NMO) is an inflammatory disease affecting the CNS (14) with similar physiopathology as MS, but is considered an autoimmune astrocytopathy. NMO is a rare disease which presents with incidence between 0.05 and 0.4/100,000 (15, 16). About 70% of the patients diagnosed with NMO shows the presence of anti-aquaporin-4 (AQP4) antibody as well as specific T-lymphocytes in the bloodstream or CSF which suggest the pro-inflammatory role of these cells (17). Importantly, the detection of anti-AQP4 antibodies is related with more severe disease (14). Recently, among seronegative patients, anti-(MOG) antibodies have been described as the pathological antibody (18). This disease has its own international consensus diagnostic criteria (19), defining the NMO spectrum disorder (NMOSD) concept (Table 2). Different MS drugs such as natalizumab or finolimob have been evaluated in NMO resulting in exacerbation of relapses (20). Immunomodulatory or immunossuppressant therapies are used for label in NMOSD (e.g., azathioprine, mycophenolate, cyclophosphamide, or rituximab) (21). Furthermore, several monoclonal antibodies are in clinical trials to evaluate their efficacy and safety, as tocilizumab, satralizumab, eculizumab, or aquapuromab for example (22). Based in the unmet need of achieving higher levels of efficacy and/or better safety profile, antigen-specific therapies are being considered as a potential treatment for MS and NMO (19).

**Table 2:** Neuromyelitis optica spectrum disorder diagnostic criteria from Ref. (23).

Diagnostic criteria for NMO spectrum disorder (NMOSD) with aquaporin-4 (AQP4)-IgG. At least 1 core clinical characteristic. Positive test for AQP4-IgG using best available detection method (cell-based assay strongly recommended). Exclusion of alternative diagnoses.

Diagnostic criteria for NMOSD without AQP4-IgG or NMOSD with unknown AQP4-IgG status. At least 2 core clinical characteristics occurring as a result of one or more clinical attacks and meeting all of the following requirements: (a) At least 1 core clinical characteristic must be optic neuritis, acute myelitis with LETM, or area postrema syndrome (b) Dissemination in space (2 or more different core clinical characteristics) (c) Fulfillment of additional MRI requirements, as applicable Negative tests for AQP4-IgG using best available detection method, or testing unavailable Exclusion of alternative diagnoses

Core clinical characteristics. Optic neuritis. Acute myelitis. Area postrema syndrome: unexplained hiccups or nausea and vomiting. Acute brainstem syndrome. Symptomatic narcolepsy or acute diencephalic clinical syndrome with NMOSD-typical diencephalic MRI lesions. Symptomatic cerebral syndrome with NMOSD-typical brain lesions.

Additional MRI requirements for NMOSD without AQP4-IgG and NMOSD with unknown AQP4-IgG status. Acute optic neuritis: requires brain MRI showing (a) normal findings or only nonspecific white matter lesions, OR (b) optic nerve MRI with T2-hyperintense lesion or T1-weighted gadolinium-enhancing lesion extending over 1/2 optic nerve length or involving optic chiasm. Acute myelitis: intramedullary MRI lesion extending over 3 contiguous segments (LETM) OR 3 contiguous segments of focal spinal cord atrophy in patients with history compatible with acute myelitis. Area postrema syndrome: dorsal medulla/area postrema lesions. Acute brainstem syndrome: periependymal brainstem lesions.

## Dendritic Cells (DCs)

**Figure 1:**
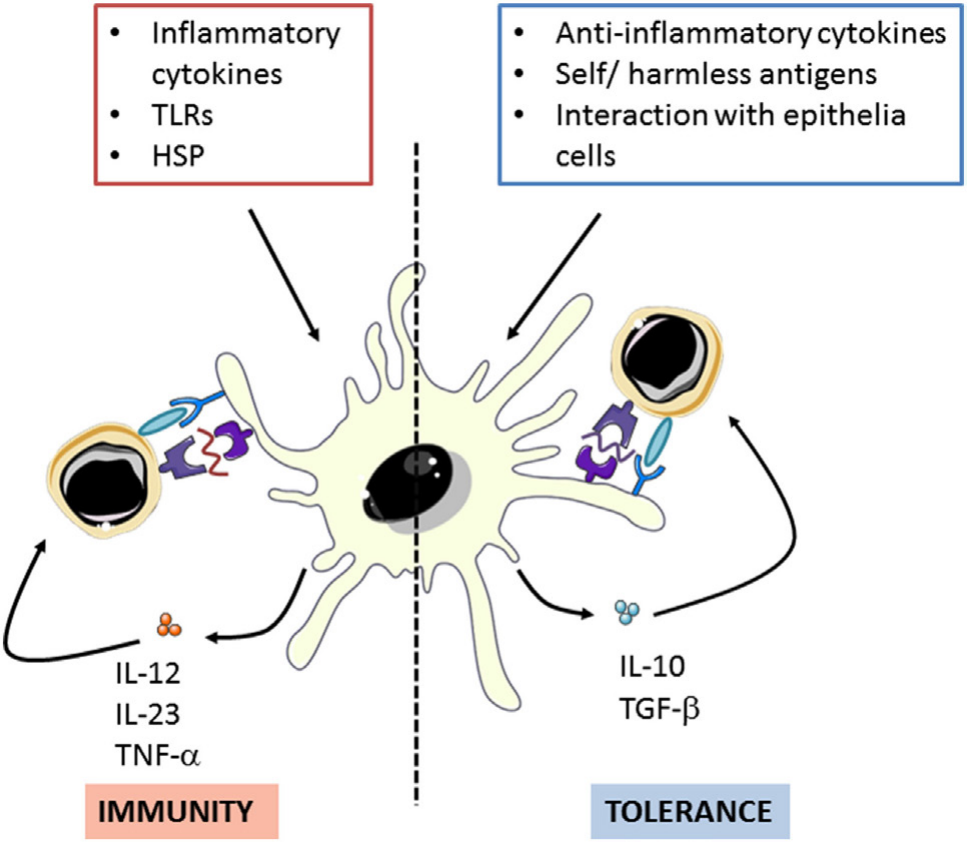
Dendritic cells can polarize immune response though promote both pro- and anti-inflammatory activities in response to different stimuli. Adapted from: O’Neil et al. (26). TLRs: toll-like receptors, HSP: Heat shock proteins.

Dendritic cells act as a link between innate and adaptive immune responses. Their main function is to capture and process exogenous antigens in the peripheral tissues to present them to T-cells after migration to the draining lymph nodes. In addition, they polarize immune responses by promoting both pro- and anti-inflammatory immune responses depending on the presence of danger signals associated to the antigens (Figure 1) (24, 25).

**Figure 2:**
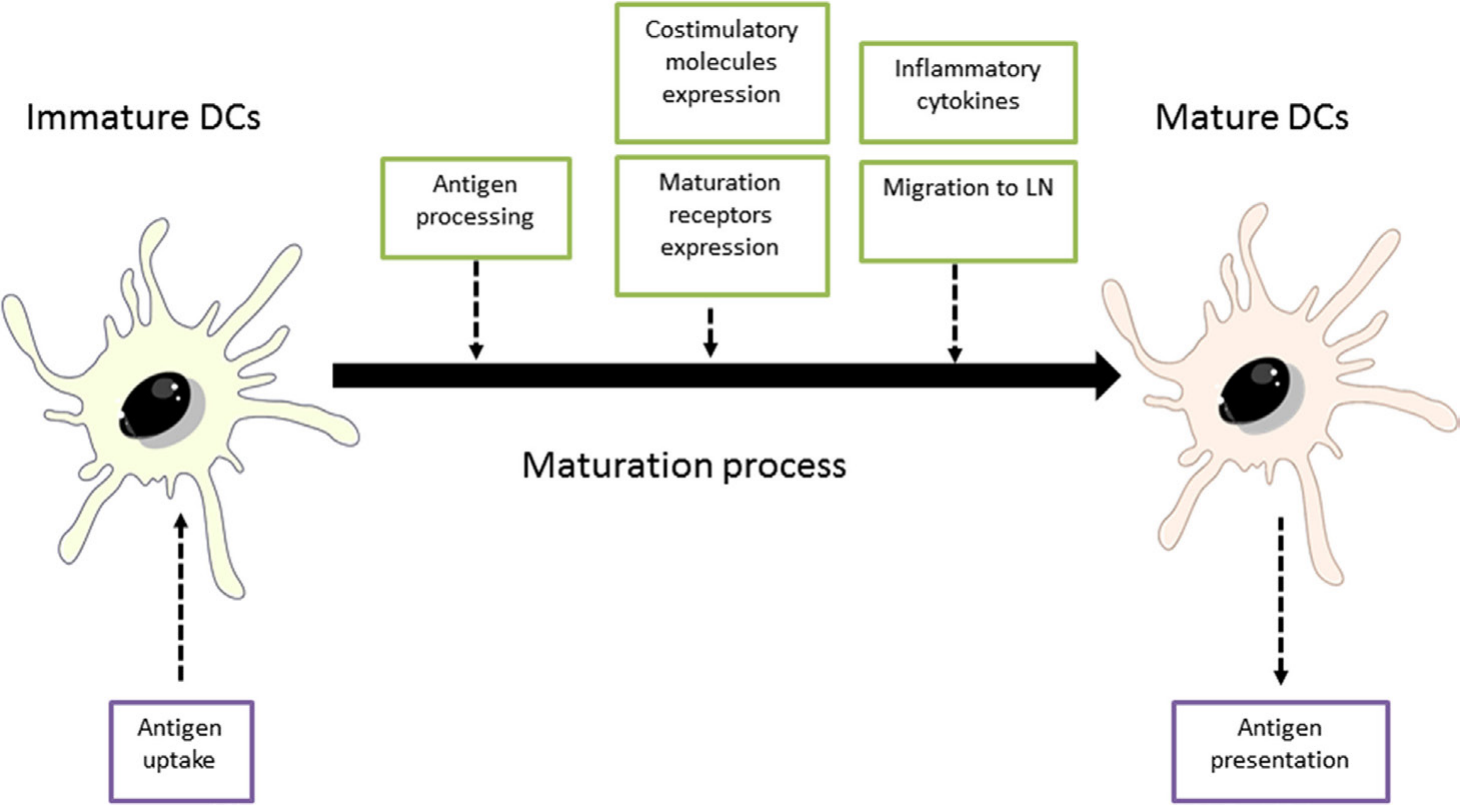
Maturation process of dendritic cells (DCs). Adapted from: Steinman et al. (29). LN, lymph nodes.

Dendritic cells are located in peripheral tissues (skin and mucosa) and remain in an immature state (iDCs) until they interact with the antigens. After cells activation, DCs initiate a maturation process in which mature DCs (mDCs) lose capacities for antigen uptake in favor of acquiring stimulatory properties for the activation of naïve T-cells and the development of effector T-cells (27). Maturation process involves different processes and physiological changes in DCs, which are illustrated in Figure 2 (28).

Due to their immunological functions and the availability of clinical-grade reagents, immunogenic DCs have been safely used in clinical trials to potentiate immune response against tumors or infectious diseases (30). However, only a few studies recently published have taken advantage of their specific tolerogenic properties to treat Type 1 diabetes, rheumatoid arthritis (RA) and Crohn’s disease patients (25, 31, 32).

## Human DCs Subsets

Dendritic cells can be sub-classified based on anatomical location, origin, and function. In humans, different DC subsets have been identified in blood, spleen and skin and in non-lymphoid tissues. Each DC subset presents different specialization in T-cell priming and induction of immune responses, although their functions can partially overlap (33).

In peripheral blood, DCs that express Human Leukocyte Antigen—antigen D Related (HLA-DR) and lineage negative fraction are divided into two main groups: conventional myeloid DCs (cDCs) and non-conventional plasmacytoid DCs (pDC). Within myeloid DCs two main subsets have been identified based on their surface marker expression: CD1c/BDCA-1 cDCs and CD141/BDCA-3 cDCs. However, recently new DC subset classification has been described (CD16 and DC5) (23). Circulating DCs represent a little fraction of total circulating peripheral blood mononuclear cells (PBMCs) as they account for less than 1% of PBMCs (24, 34).

In the skin two different subsets of DCs can be found. Langerhans cells (LCs) which contributes to immune surveillance and CD14 DCs, which are involved in tolerance induction (35, 36).

**Figure 3:**
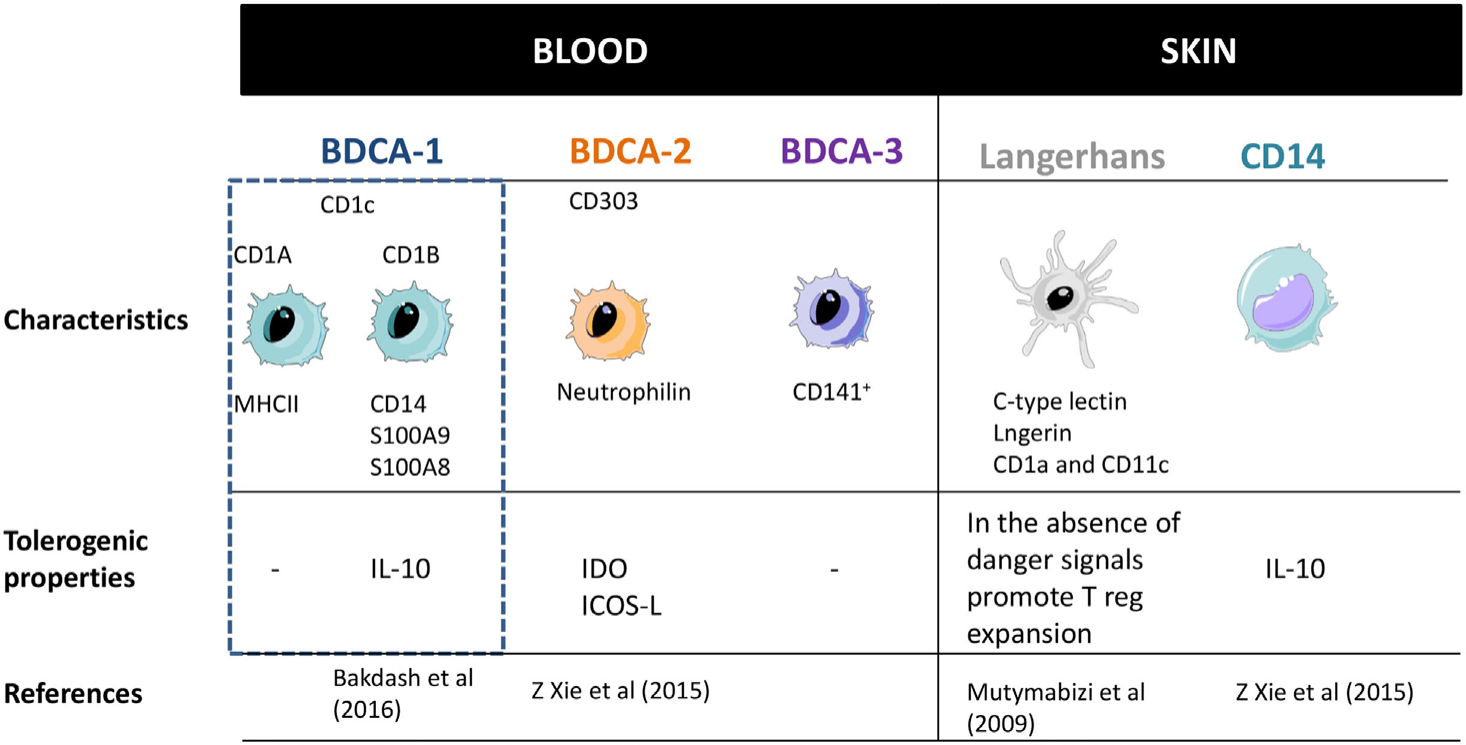
Dendritic cells subsets classification and their main properties. Adapted from: Cohn et al. (37). PRRs, pattern recognition receptors.

From all the different DC subsets above mentioned, the BDCA-1, pDCs, LCs, and CD14 have been described to generate both immunogenic and suppressive functions (Figure 3). BDCA-1 have the capacity to produce IL-10 in response to *E. coli* and potentially contribute to suppress immune responses. Recently, a particular subset of BDCA-1 (BDCA1-CD14^+^) has been shown to act as immunosuppressive cells in certain types of tumor environment and may hamper anti-cancer DCs vaccines (37, 38) Moreover, in an steady state, pDCs are able to induce tolerogenic immune responses by inducing T-cell anergy and promoting T-reg cells development. They have been found to be infiltrated in tumors activating Tr1 cells (33, 39, 40). LCs, apart from respond to intracellular pathogens and viruses under inflammatory conditions are in charge to maintain epidermal health and tolerance to commensals from the skin, while retaining the ability to respond to selected pathogens (40–42). Finally CD14 DCs also have the ability to generate T-regs through the elevated IL-10 production (43, 44).

To sum up, BDCA-1, pDCs, LCs, and CD14 have been shown to present immunoregulatory effects. However, deeper characterization of this tolerogenic profile and mechanisms needs to be performed.

## Tolerogenic DCs (Tol-DCs) and Mechanisms of Tolerance Induction

**Figure 4:**
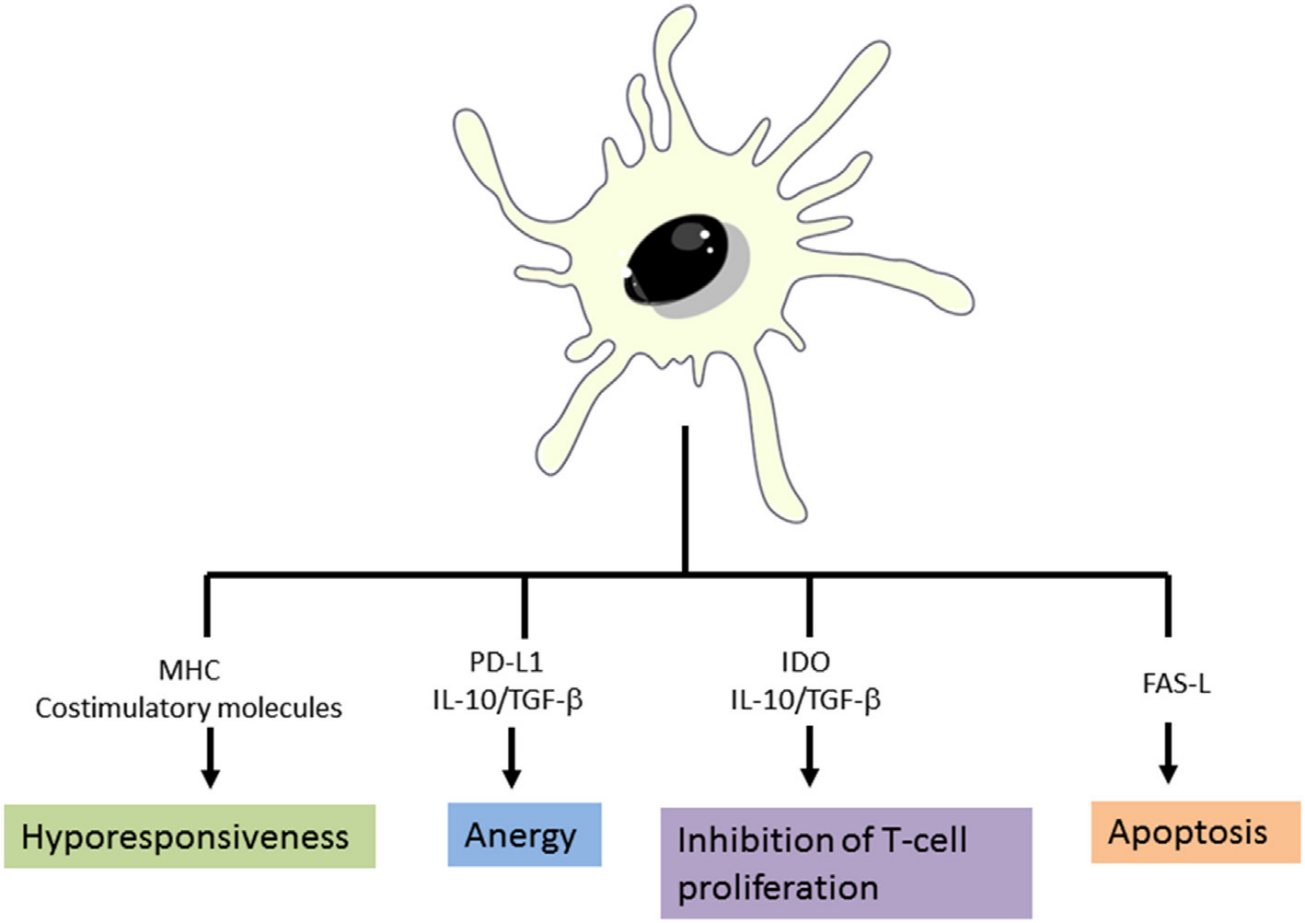
Mechanisms of tolerance induction by dendritic cells. Adapted from: Cabezón et al. (30).

As described in the previous section, DCs play a crucial role in the initiation of immune responses and also in maintaining the immune tolerance. DCs present both foreign antigens as well as endogenous antigens derived from tissues. For this reason, the immune system is able to distinguish between innocuous and harmful antigens to avoid autoimmune or undesired immune responses (45). Several studies point that a key factor for DCs to initiate immunity or tolerance is the maturation stage of DCs (25). It is generally accepted that in absence of danger signals provided by infection or inflammation, DCs remain in an immature state which will induce tolerance by deleting or inducing apoptosis of self-antigen-specific T-cells (25, 46). However, other several mechanisms to explain how DCs induce tolerance have been proposed. Some authors have reported that low expression of MHC molecules and co-stimulatory receptors on DC surface fail to stimulate T-cells sufficiently, thus resulting in T-cell anergy (47–49). Currently, it has been demonstrated that the expression of single immunoglobulin IL-1 related receptor, which is lower in iDCs, has a role in maintain low levels of costimulatory molecules and in the regulation of T-reg cell expansion (50). Furthermore, it is well established that the expression of certain molecules such as PD-L1 rather than promote activation signals to T-cells, they induce T-cell anergy (28, 51, 52). Moreover, some authors demonstrated that suboptimal antigen presentation, together with indoleamine 2,3-dioxygenase (IDO) or Fas-L (CD95L) expression by DCs leads to inhibition of T-cell proliferation and T-cell deletion, respectively. Finally but not the least, the production of the potent anti-inflammatory cytokine IL-10 by DCs is crucial for peripheral tolerance induction. IL-10 acts on a wide variety of immune cells and it has been clearly involved in T-reg as well as Tr1 induction (38). In the steady state, peripheral T-reg cells rise from peripheral CD4^+^CD25^−^FOXP3^−^ T cells that are exposed to antigen in the presence of transforming growth factor-β as well as IL-10 without IL-6 or IL-1β, which promotes the up-regulation of FOXP3 (17) (Figure 4). Recent developments carried on by Agrawal et al., have shown that C-lectin receptor (CLEC-2) upregulation in DCs, is associated with T-reg induction. Moreover, they have also described that platelet growth factor is able to induce IL-10 production by DCs and in consequence T-reg cell induction (53).

In consequence, major efforts have been focused on *in vitro* generation of Tol-DCs. In this regard, different immunosuppressive drugs, such as corticosteroids, cyclosporine, tacrolimus, rapamycin, deoxyspergualin, vitamin D3 (vitD3), mycophenolate mofetil, and sanglifehrin A, have been successfully used to modulate DCs differentiation and function. Thus, several protocols that include the generation of monocyte-derived DCs in the presence of corticosteroids and a defined maturation cytokine cocktail (including TNF-α, IL-1β, IL-6, and PGE2) or lipopolysaccharide (LPS) activation in order to boost their tolerogenic properties, have been described to generate Tol-DCs *in vitro* (54, 55).

**Figure 5:**
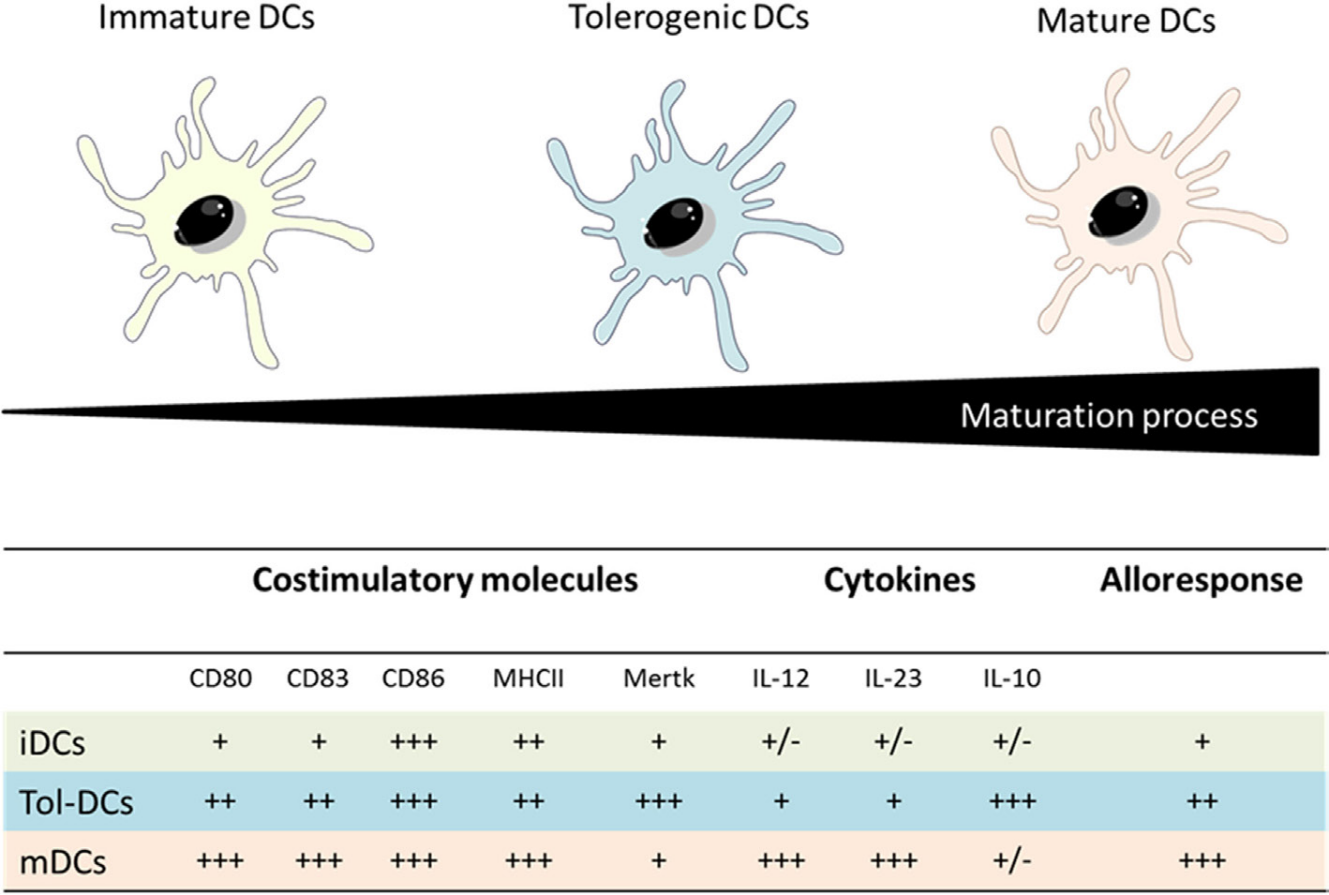
Comparison between iDCs, tolerogenic DCs and mature DCs properties. Adapted from: Hubo et al. (59).

Tolerogenic DCs present an intermediate phenotype between iDCs and mDCs regarding costimulatory molecules, a pronounced shift toward anti-inflammatory versus pro-inflammatory cytokine production (high amounts of IL-10 versus low levels of IL-12p70 and IL-23) and a reduced capacity to stimulate T-cells response. In addition Tol-DCs present an increment of IL-10 production upon Gram-negative bacterial interaction which represents a relevant factor to induce tolerance due to the potent anti-inflammatory role of IL-10 (Figure 5) (56–58).

The role of *in vitro* generated Tol-DCs as potential immunomodulatory and immunosuppressive agents have been evaluated by different groups (44, 60, 61). The first experimental data to objectify the potential of human Tol-DC to induce tolerance in MS, was the induction of T-cell hyporesponsiveness by Tol-DC from MS patients. The results obtained shown that only Tol-DCs (vitD3) derived from RRMS patients, induced hyporesponsiveness in autologous antigen-specific T-cells restricted to myelin-derived peptides and produced higher levels of IL-10 and reduced levels of TNF-α compared to healthy controls, making the tolerogenic potential of these autologous Tol-DCs may be an effective tool to re-establish tolerance in RRMS patients and set up the basis for the ongoing clinical trials (62). In addition, a critical consideration for Tol-DC application in immunotherapy is the phenotype stability once the cells are injected into the patients. It has been demonstrated that *in vitro* generated Tol-DCs have a stable tolerogenic profile after LPS stimulation as they produce higher amounts of IL-10 and as well as they are able to induce antigen-specific T-cell hyporesponsiveness (58, 63).

In summary, Tol-DCs generated *ex vivo* using immunosuppressive agents, induced T regulatory cells through different mechanism such as lower expression of co stimulatory molecules, expression of inhibitory receptors and IL-10 production.

### Tol-DC Therapy in the Animal Model of MS

Animal models are the first step in the development of new therapies, and antigen-specific therapies are not an exception to this rule. Over the past several decades animal models have been used to understand different aspects of human MS. There are three different animal models of MS that are the most commonly used: (1) the experimental autoimmune encephalomyelitis (EAE), (2) viral induced models, and (3) toxin-induced models of demyelination (6).

In addition to the *in vitro* demonstration of the capacity of Tol-DC to induce immune tolerance, the role of Tol-DCs has been evaluated in the EAE model. The critical role of mDCs and pDCs in the chronic pathogenesis of EAE in Lewis rats described by Miller and colleagues makes this model extremely relevant to study positive and negative regulatory pathways involved in MS and other chronic autoimmune diseases (64). Wang et al. demonstrated the involvement of CD11b^+^ and CD11c^+^ DCs in the generation of both T-regs and Tr1 cells, by depleting DCs they observed that tolerance effect disappeared (65). In consequence, the induction of DCs with a regulatory profile is a key mechanism underlying auto antigen-induced tolerance (64). It is interesting to highlight that studies performed in EAE induced in Lewis rats demonstrated that the maturation state as well as the route of administration influence on the induction of tolerance by these DCs which is in concordance with the *in vitro* performed studies (65, 66). Moreover, different authors have described that the administration of Tol-DCs generated with different immunosuppressive agents such as vitD3 or estriol induced a decrease of the incidence of the disease as well as they promoted the induction of regulatory T-cells though higher levels of IL-10 production (63, 67).

In addition, other authors have performed comparisons regarding the use of immunosuppressive oral drugs such as vitD3 and (for 20 days after EAE induction) or pretreating DCs before EAE induction. The results obtained were similar in both cases: significant improvement of clinical severity and an increase of regulatory CD4^+^ Foxp3^+^ cells and increased IL-10 levels in lymph nodes from treated animals suggesting that DCs are the main target of tolerogenic effect of vitamin D. Some studies pointed out that in the absence of DCs during the priming process of autoreactive T-cells leads to a unidirectional deficiency of cell generation which results in a fulminant attack against CNS (65, 66, 68). Different studies using DCs to induce tolerance have been performed in EAE animal models of mice and rats and they are summarized in Table 3.

**Table 3:** Summary of tolerogenic DCs therapy in animal models (65, 67, 69, 70).

Animal model	Dendritic cells injected	Route of administration	Reference
EAE in C57BL/6 mice	1 × 10^6^	Intravenous	Leng et al. (67)
EAE in C57BL/6J mice	1 × 10^6^	Intravenous	Mansilla et al. (63)
EAE in C57BL/6 mice	1–2 × 10^6^ to 8–10 × 10^6^	Intravenous	Papenfuss et al. (67)
EAE in C57BL/6 mice	5 × 10^5^	Subcutaneous or intraperitoneally	Aghdami et al. (71)
EAE in Lewis rats	2 × 10^6^	Subcutaneous or intravenous	Zhang et al. (72)
EAE in Lewis rats	1 × 10^6^	Subcutaneous	Xiao et al. (66)

*EAE, experimental autoimmune encephalomyelitis*.

In addition, Tol-DCs have also been generated for another disease models such as type I diabetes T1D by using a combination of both dexamethasone and vitD3. This generated Tol-DCs presented a stable phenotype and a high capacity to induce T-reg cells (73). Moreover, other protocols, such as DC treatment with CD40, CD80, and CD86 antisense oligo nucleotides or even low doses of GM-CSF has also been reported although in some cases partial loss of tolerance have been reported.

The critical part is that after being culture, all generated Tol-DCs have to present different characteristics: (a) low levels of co stimulatory molecules, (b) stability when challenges with maturation stimuli and also produce IL-10, (c) lower activation of T-cells (73).

Overall, different protocols for Tol-DCs in preclinical studies has been shown to be beneficious to treat different autoimmune diseases, in particular for EAE induction the use of vitD3 or corticosteroids is the most extended.

### Therapeutic Application of Tol-DCs in Type I Diabetes, RA and Crohn’s Disease

Following the encouraging results obtained from different *in vitro* and preclinical studies in animal models, Tol-DCs are revealed as a promising therapy for autoimmune diseases and transplantation (32). Consequently, in 2011, the first phase I clinical trial with Tol-DCs was conducted at the University of Pittsburgh. The trial enrolled 10 insulin-dependent diabetic patients, and administrated control DCs to three patients and immunosuppressive DC (iRNA for CD40, CD80, and for CD86) to seven patients. The treatment was safe and well tolerated. There were no changes in insulin requirements, hematology assessments or blood immune cell population levels in both groups, showing a slight increase of CD4^+^CD25^+++^ FoxP3^+^ T cells in immunosuppressive DC group. All treated patients had normal immune responses to vaccination and alloantigen stimulation *in vitro* (74). Thus, a double-blinded, placebo-controlled cross-over phase II trial is planned to start in Diabetes mellitus type 1 in 24 patients with a recent onset of the disease, inducing tolerability of DC with antisense DNA targeting CD40, CD80, and CD86 (NCT02354911).

Among autoimmune arthritis, two trials have been published recently. In the first one, a unique intradermal administration of “Rheumavax” (autologous DCs modified with a nuclear factor κb inhibitor exposed to 4-citrullinated peptide antigens), was studied in a phase I clinical trial of RA patients. They observed a significant increased ratio of regulatory to effector T cells and a reduction of IL-15, which is a relevant pro-inflammatory cytokine. Moreover, in a more clinical level they found a decrease of DAS28 which is a clinical scale for RA severity together with no disease flares (75). Furthermore, in 2017, results from AUTODECRA trial (NCT01352858) came out resulting a safe and well tolerated therapy with no target knee flares, but with no significant clinical and immunomodulatory changes in serum (76).

Importantly, other clinical trials have been recently reported in other autoimmune diseases such as Crohn’s disease. In Crohn’s disease, our institution conducted a phase I clinical trial to demonstrate the safety of intraperitoneal administration of autologous Tol-DCs in refractory patients. The immune monitoring studies showed an increase of circulating T-regs and a decrease of IFN-γ production after T-cell activation (31). Regarding organ transplantation, two trials are ongoing. A phase I clinical trial, open-label and non-controlled, in liver transplantation is aimed to assess the safety of Tol-DCs therapy in this type of patients (NCT03164265). The ONEatDC study, aims to assess if Tol-DC administration before renal transplantation is beneficial to reduce immunosuppression needs (NCT02252055).

Overall, the encouraging results obtained in above mentioned clinical trials, of an increase immunosuppressive activity, drawn Tol-DCs as a potential tool to modulate autoinflammatory diseases in the coming years.

## Antigen-Specific Therapies in MS and NMO

In the recent years, several strategies to modulate antigen-specific T-cells have been evaluated in therapeutic clinical trials for patients with MS and NMO. Among the advantages to use antigen-specific therapies, they lack of general immunosuppression and its side effects as infections and cancer, as well as the lack of metabolic activity that activates self-reactive T cells, the induction of tolerance to a specific antigen without changing the general immunity (77). The use of DC to induce immune tolerance is also pursuit in patients with MS and NMO. In this sense, a phase I trial to assess the safety of Tol-DC in MS and NMO patients in an ascending dose of intravenous administration of the DCs (NCT02283671) has been performed at our institution and the results are under evaluation. In addition, two more clinical trials are ongoing (NCT02618902) and (NCT02903537), which will provide precious information about safety, modulation of immune response and clinical efficacy.

Several approaches to induce antigen-specific tolerization have been evaluated as DNA vaccination of myelin protein, peptides inoculation, altered peptide ligand (APL) administration to modify TCR recognition, autologous myelin-reactive T cells administration, HLA/MOG recombinant construct administration and autologous PBMCs coupled with myelin-peptides administration, Tol-DCs with myelin-peptides administration (78). Specifically, myelin-peptides approaches are based in a myelin relevant immunodominant peptide administration, like administration of the synthetic peptide itself like MBP, MOG, or PLP, administration of APL or the administration of a region of TCR-peptide complex.

Antigen-specific therapeutic approaches have been demonstrated in the majority of the phase I clinical trials to be safe and well tolerated. However, a trial conducted at NIH with APL induced disease exacerbation and the trial was stopped due to safety issues (79). The concept of APL is based in the administration of modified peptides by introducing some amino acids in substitution in specific positions relevant to link with the TCR, but without changing the MHC binding part. This strategy is aimed to inhibit the inflammatory T cell response, as acts as partial agonist or as antagonist. A phase II trial using MBP_83–99_ was interrupted as three out of eight participants presented relapses during the clinical trial, that were considered as inflammatory activation as MRI controls showed disease worsening, and this was correlated with MBP specific T cell expansion in blood and CSF samples (80). Two more trials with APLs were done afterward, without objectifying exacerbations of the disease activity (81).

DNA vaccination aims to induce tolerance using heterotopic expression of some antigens, for example using whole human MBP protein. The BHT-3009 molecule is a union of the whole MBP molecule, a human cytomegalovirus promoter and an altered plasmid. In two clinical trials it was demonstrated safe and gadolinium-enhancing lesions were fewer in the treated groups comparing with placebo groups; although, there were significant improvement in clinical outcomes. Immunologically, a decrease in IFN-γ production and T cell proliferation by MBP, PLP, and MOG specific T-cells was observed (82). In another trial, reduction of autoreactive T cells was demonstrated with this approach, creating a proof of concept of the possible efficacy of DNA vaccination (80).

The vaccination with T-cell consists in the administration of activated and irradiated MPB-specific T-cell lines and clones (attenuated autologous T-cells). Phase I and phase II clinical trials have been done, with no relevant side effects, but without significant clinical improvement in treated group comparing with placebo group (83).

Other antigen-specific tolerization approach studied in MS was the antigen-coupled cell tolerance, based on inactivated autologous PBMCs chemically linked with myelin relevant peptides. After proving reduction of onset and severity as well as preventing epitope spreading in EAE, this approach was evaluated in humans. In 2013, a phase I clinical trial (ETIMS trial) was published where antigen-specific tolerance induced with inactivated PBMCs coupled with six immunodominant myelin-peptides was safe, with some immunological promising results to objectify clinical significance (78). Significant advantage of this approach is that the tolerization to several myelin relevant peptides derived from three different antigens (MBP, MOG, and PLP) simultaneously is aimed to prevent the epitope spreading situation.

To synthetize, there are different antigen-specific therapies that have been asses in MS patients. The majority has been presented as safe and well tolerated with encouraging data regarding the clinical benefits.

## Conclusion and Future Perspectives

Antigen-specific tolerance in autoimmune diseases is a therapeutic approach that is currently been evaluated in MS and NMO as well as in other autoimmune diseases. Different reports have demonstrated that DCs are powerful therapeutic tools to modify the immune response and restore the immune tolerance in animal models and in preclinical data. Most importantly, the use of Tol-DCs in clinical trials is being safe in several phase I clinical trials (type I Diabetes, RA and Crohn’s disease) showing in some of the studies promising clinical and immunomodulatory results.

In MS several reports have revealed the therapeutic effect of Tol-DCs in ameliorating EAE in animal model. These results highlight the importance of DCs in the homeostasis control and open new avenues for an innovative therapeutic indication for human patients. A major challenge is to translate all these results obtained in animal models to humans. For that reason, it will be crucial to correlate clinical efficacy with modulation of immunological parameters and also to define the optimal administration route, dose of cells, tolerogenic treatments and the potential tolerogenic effect of circulating DCs.

From the studies conducted so far, several important considerations have been raised, application of Tol-DCs in humans is safe and well tolerated without remarkable side effects and showing promising immunological and clinical results. However, phase II and/or III clinical trials including control (placebo) group will bring some light about the clinical efficacy of this therapy in MS/NMO patients. In addition, more studies are needed to evaluate the real effectiveness and the possibility to use Tol-DC as a real treatment for autoimmune diseases.

## Author Contributions

GF-G, IZ, and RC wrote the manuscript and designed figures. PV and DB-R revised the manuscript.

## Conflict of Interest Statement

PV is an employee of Genentech. The remaining authors declare that the research was conducted in the absence of any commercial or financial relationships that could be construed as a potential conflict of interest. The reviewer IL-P and handling Editor declared their shared affiliation.

## Abbreviations

APCs, antigen presenting cells; APL, altered peptide ligand; AQP4, aquaporin-4; CNS, central nervous system; DCs, dendritic cells; Mo-DCs, monocyte-derived DCs; MS, multiple sclerosis; NMO, neuromyelitis optica; PPMS, primary-progressive MS; RA, rheumatoid arthritis; RRMS, relapsing-remitting MS; SPMS, secondary-progressive MS; Tol-DCs, tolerogenic DCs.
